# General palliative hospital care – a Danish nationwide survey of organization and clinical practice

**DOI:** 10.1186/s12904-026-02005-3

**Published:** 2026-01-28

**Authors:** Heidi Bergenholtz, Tine Ikander, Ida Fritsdal Refer, Tina Broby Mikkelsen, Kirstine Skov Benthien

**Affiliations:** 1 REHPA, The Danish Knowledge Center for Rehabilitation and Palliative Care, University of Southern Denmark, Nyborg, Denmark; 2 Zealand University Hospital, Roskilde, Denmark; 3 Department of Respiratory Medicine and Endocrinology, Copenhagen University Hospital, Kettegaard Allé 30, Hvidovre, 2650 Denmark; 4 Department of Clinical Medicine, University of Copenhagen, Copenhagen, Denmark

**Keywords:** Palliative care, Palliative medicine, General palliative care, Hospital, End-of-life

## Abstract

**Background:**

Most patients end their life with general palliative care offered by healthcare providers in non-palliative care focused/specialized departments. The state of how this care is organized and adherence to the Danish board of health recommendations is largely unknown.

**Objective:**

The aim of this study is to describe the organization and clinical practice for general palliative care in Danish hospitals and to explore the association between attitudes and clinical practice.

**Design:**

The study is a cross-sectional survey of hospital departments in Denmark with a partial comparison to a survey one decade earlier. Analyses were descriptive and logistic regression.

**Participants:**

The questionnaire was sent to one randomly assigned chief of all 360 clinical hospital departments in Denmark.

**Key results:**

The response rate was 73%. Physical symptoms were addressed by 96% and spiritual problems by 56%. The proportion of departments that prioritized resources for general palliative care increased from 24% in 2013 to 51% in 2023. Assessment of needs for general and specialized palliative care was mostly based on a general appraisal. In multivariable logistic regression analyses, the medical departments were more likely to perform needs assessment than the surgical departments, and the attitude towards systematic needs assessment was associated with the clinical practice and planning end-of-life care in the department. The responsibility for planning end-of-life care was regarded as shared among several stakeholders.

**Conclusions:**

General palliative care was focused on physical symptoms and assessment of target group and needs were rarely performed systematically despite national recommendations. Clinical practice was associated with specialty and attitudes.

**Supplementary Information:**

The online version contains supplementary material available at 10.1186/s12904-026-02005-3.

## Background

Providing general palliative care within the hospital setting presents a unique challenge. Hospitals are inherently paradoxical environments, simultaneously focused on life-saving interventions and end-of-life care [1]. General palliative care is delivered by healthcare professionals in non-palliative care focused departments and is distinct from specialized palliative care, typically delivered by hospices, dedicated palliative care units, and specialized teams [2].

In a Danish context, data from the National Cause of Death Register in Denmark indicates that, on average, 35% of all deaths nationwide occur within hospitals [3]. Despite its potential significance, access to general palliative care remains unequal across populations, with certain groups experiencing barriers that limit their ability to receive these essential services [1, 4, 5]. Patients with cancer receiving specialized palliative care constitute a considerably higher proportion (46%) compared to patients succumbing to other illnesses (3%) [6]. In a cross-section, 22% of hospitalized patients died within one year of admission [7]. These results suggest that many inpatients may be approaching the end of life and could benefit from general palliative care. Furthermore, leading causes of death remain long-term non-communicable diseases such as cardiovascular disease and chronic lung illnesses [8], and most treatment trajectories should, therefore, allow the possibility of early general palliative care.

The most recent mapping of the structural and organizational aspects of general palliative care in Danish hospitals was conducted in 2013 [9] but since then, several national documents focusing on early palliative care, organization, and prioritization have been published [10, 11]. The government administration stresses that healthcare inequity should be avoided, the target group for palliative care defined by life-threatening disease [12] should be systematically identified, and the need for palliative care systematically assessed [11]. Since palliative care specialists only have knowledge about the patients in their own clinical care, the target group identification and needs assessment will take place in general palliative care. Access to palliative care services for inpatients can meet various barriers, including having a non-cancer condition and the focus on cure in hospitals [1], which highlights the need for knowledge about needs assessment practices. This process is crucial for delivering appropriate and tailored palliative care services. In summary, the overall state of general palliative care in Danish hospitals remains unknown. This study aimed to describe general palliative care in Danish hospitals including how general palliative care is organized within hospitals, how national recommendations are translated into practice and how leadership attitudes influence needs assessment and end-of-life planning.

## Methods

### Design

This study was a nationwide survey of general palliative care as reported by department heads in Denmark. The study is cross-sectional and includes a partial comparison to a survey conducted in 2013 [9]. The 2013-survey focused on strategy, but it also included questions about resources, which were repeated in the present study.

### Setting

The Danish healthcare system is universal and tax-funded. The hospitals are managed by five regions with varying population characteristics, rural/urban composition, and separate political management. Hospital department chiefs are second level managers, and they are responsible for the quality of clinical care and manage resource allocation for skill training.

### Human ethics and consent to participate declaration

The informants have consented to participate, and the data collection is stored according to the General Data Protection Regulation and approved by the Region of Southern Denmark (Acadre case no. 23/3979). The study type does not require ethics committee approval by Danish law.

### Consent to publish declaration

Not applicable.

### Questionnaire

The resource allocation items from the 2013 survey [9] were repeated in 2023 and the questionnaire was further expanded with several items about needs assessment and tools that were developed during the last decade. In the next round, the questionnaire was elaborated by a group of stakeholders from patient organizations, healthcare professionals, and decision-makers. Finally, the questionnaire was adjusted using cognitive interviewing [12] in which three respondents in the target group elaborated on their understanding of the questions and the questionnaire was revised accordingly. The final questionnaire included items about department characteristics, target group, organization, clinical practice, end-of-life care, advance care planning, needs assessment and attitudes towards screening defined as “how favorably or positively one is predisposed towards using a particular evidence-based practice” [13], and use of tools. The questionnaire also included the four dimensions of palliative care as defined by the World Health Organization: the physical, psychological, social, and spiritual dimensions [14]. The survey included definitions of general and specialized palliative care. In case the department included a specialized palliative care unit, the respondents were instructed to report only on the general palliative care performed by other units. Questions employed categorical options, some with one answer and some, where multiple answers were possible. This article includes selected results from the survey that totaled 50 items.

### Procedure

The questionnaire was sent in May 2023 to all clinical hospital departments in Denmark excluding supportive departments such as radiology and microbiology. Since most departments have a chief nurse and a chief physician, the survey was sent to a randomly selected chief. In case of lacking response, two reminders were sent, then the survey was sent to the other chief, and finally a last reminder was sent. Only one questionnaire was completed by each department. The questionnaires were collected using SurveyXact. All respondents received questions about department characteristics. The target group was further selected through two filter questions. The first was: “Does the department have in- or outpatients with life-threatening, incurable disease?“. The questionnaire was finished for No-responders, while Yes- and Unsure-responders received questions about needs assessment and their attitude towards screening and use of tools. The second filter question was, “Does your department ever come to provide general palliative care?“. The questionnaire was concluded for No-responders to that question. Due to the filter questions and the possibility of concluding the survey at any time, the number of completed items varies throughout the analyses. Furthermore, a confirmatory response to some questions activated other questions to elicit further details. The procedure was similar to the survey performed in 2013.

### Data and analyses

Covariates included yearly inpatient admissions (< 2000, 2000+, and unsure), yearly outpatient contacts (< 10.000, 10.000+, and unsure), region of residence, and types of specialties (surgical, medical and others including mixed specialties). Explanatory variables about attitudes were dichotomized in agree or strongly agree vs. agree partially or disagree or disagree very much. Outcome variables were dichotomized into no or do not know vs. yes with any method. The data were summarized with frequencies and analyzed using crude and multivariable logistic regression analyses with SAS 9.04.

## Results

The survey was sent to 360 departments of which 262 completed all the initial items about department characteristics and the first filter question – a response rate of 73%. Non-response ranged from 23 to 33% between the five regions and from 8 to 53% between hospitals with more than 10 departments. The number of departments who confirmed to have patients with a life-threatening, incurable disease was 225 (86% of responders). For the target group of departments with patients with a life-threatening disease who provided general palliative care, the response rate for single items varied from 94 to 100%. The flowchart is presented in Supplementary Figure S1.

The characteristics of participating departments are described in Supplementary Table S1. With anesthesiology and intensive care combined, cardiology and endocrinology were the specialties included most often and ten departments included sections with specialized palliative care. Respondents were instructed to exclude information from specialized palliative care and focus on the sections with general palliative care. Departments with 2000 + inpatient admissions per year accounted for 39% of the respondents.

In 2023, 104 of 203 departments (51%) had prioritized resources for general palliative care such as skill training, staff, and physical surroundings. This marks an increase from 24% in 2013.

The reported relief of symptoms and problems within the four dimensions of palliative care and the documentation in patient records is demonstrated in Supplementary Figure S2.

The four dimensions in palliative care were addressed with some variation as 96% of the departments reported relieving physical symptoms, and 56% reported relieving spiritual problems. The symptoms and problems were relieved more often than it was documented in the patient record.

36% of the departments used screening tools to identify patients with a life-threatening disease, 45% said no, and 19% were unsure. The most common screening tools were the surprise question (Would you be surprised if the patient died within 6–12 months? ) (13%) and disease-specific criteria (13%). Palliative care practices were more likely performed in medical departments compared to surgical departments (see supplementary table S2).

Table 1 describes the association between attitude towards the use of assessment tools and the reported use of those tools and the practice of planning the end of life in cooperation with the patient.

**Table 1:** Association between attitudes towards palliative care and palliative care practices

			Crude		Adjusted*	
Explanatory variable	Outcome	n	OR	95% CI	OR	95% CI
Agreement with the clinical relevance of use of screening tools to identify the target group	Use of screening tools	193	6.423	(3.305–12.484)	6.880	(3.297–14.360)
	Planning end-of-life care together with the patient	190	1.757	(0.978–3.155)	1.892	(0.990–3.616)
Agreement with the clinical relevance of systematic assessment of needs for general palliative care	Assessment of needs for general palliative care	213	3.118	(1.755–5.540)	2.818	(1.537–5.164)
	Planning end-of-life care together with the patient	190	2.571	(1.413–4.677)	2.613	(1.372–4.976)
Agreement with the clinical relevance of systematic assessment of needs for specialized palliative care	Assessment of needs for specialized palliative care	212	3.262	(1.753–6.071)	2.721	(1.395–5.307)
	Planning end-of-life care together with the patient	190	2.711	(1.496–4.914)	2.366	(1.262–4.435)

*Adjusted for region, yearly inpatient admissions, yearly outpatient contacts, and type of specialty

The reported clinical practice for screening for life-threatening diseases, assessment of the need for palliative care and planning the end of life were associated with the attitude of the department as described by one of the department chiefs. The multivariable analyses were adjusted for region, yearly inpatient admissions, yearly outpatient contacts, and type of specialty but these factors had little impact on the results.

Of the 195 departments who reported whether they screened for a life-threatening disease, 7% reported always doing so, and 18% reported often doing so. Of the 213 departments that reported whether they assessed patients’ need for general palliative care, 8% reported always doing so and 30% often. Finally, 5% of 212 departments always assessed patients’ need for specialized palliative care and 36% often did so.

The methods used for needs assessment are described in the following Table 2.

**Table 2:** Assessment of needs for general and specialized palliative care

	‘Does the department assess patients’ need for *general* palliative care?’		‘Does the department assess patients’ need for *specialized* palliative care?’	
	*n*	%*	*n*	%*
Yes, with a general appraisal	89	42	113	53
Yes, with open questions (such as ‘How are you doing?‘)	61	29	51	24
Yes, with the questionnaire EORTC QLQ-C15-Pal	33	15	32	15
Yes, with another screening tool	7	3	8	4
Yes, with another patient-reported questionnaire	6	3	5	2
Yes, with a screening tool completed by healthcare professionals	9	4	3	1
No	33	15	24	11
Unsure	37	17	23	11
Not relevant	11	5	18	8

*More than one response was allowed, and the total is more than 100%. There were 14 missing responses about general palliative care and 15 missing responses about specialized palliative care

The most common methods for needs assessments were a general appraisal of the patients’ condition and the use of open-ended questions such as ‘How are you doing?’. Of 197 departments, 67% reported always or often referring patients to specialized palliative care when relevant.

**Fig. 1:**
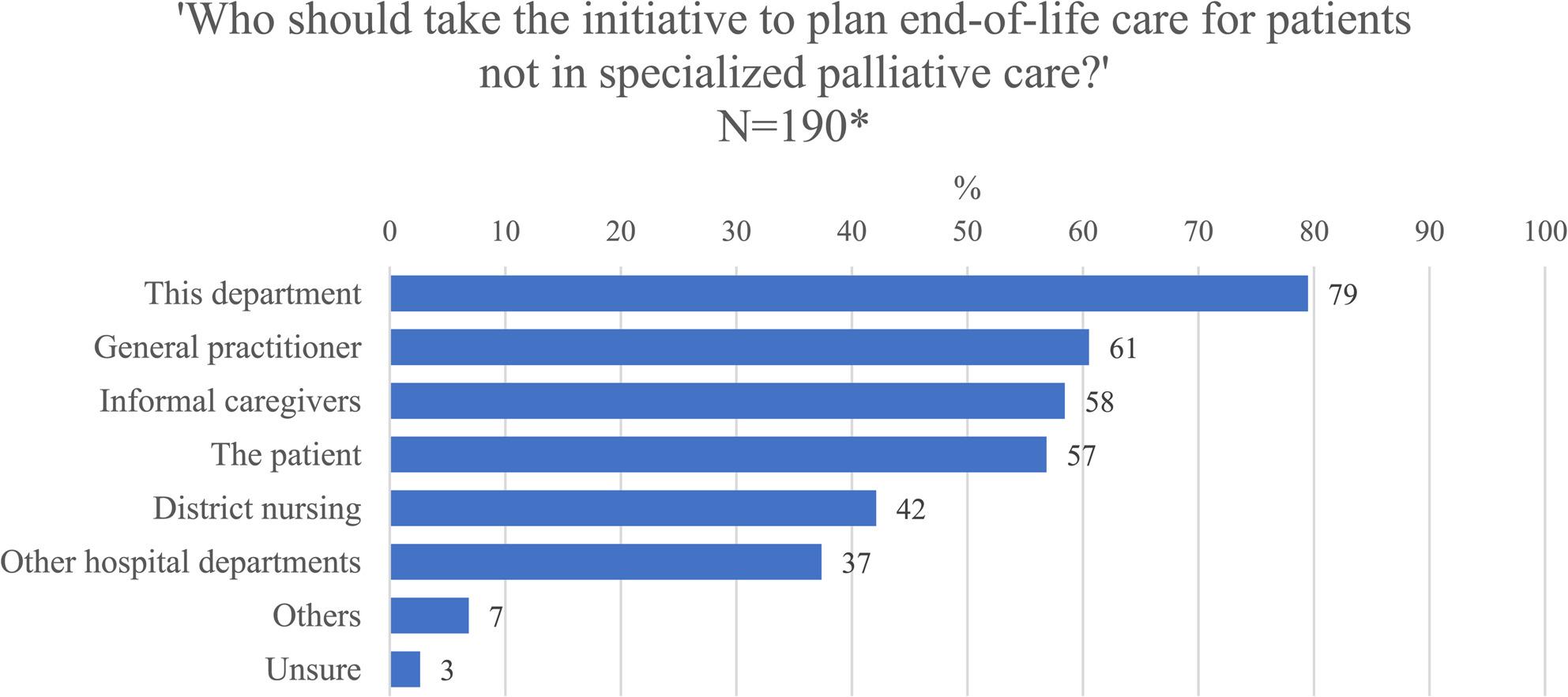
Responsibility for initiating end-of-life care planning. *More than one response was allowed, and the total is more than 100%. There were 13 missing responses

For dying patients, 83% of departments reported always or often initiating general palliative care but only 42% initiated general palliative care for patients with a life-threatening disease, who are not immediately dying. Finally, the perception of responsibility for initiating end-of-life care planning is described in Fig. 1.

As demonstrated, 79% of the departments perceived themselves as responsible for taking the initiative, 61% perceived the general practitioner as responsible, 58% the informal caregivers, and 57% the patient.

## Discussion

### Main findings

This study described the general state, organization, and clinical practice of general palliative care in Danish hospitals. With little opportunity for international comparison, we were able to indicate the progression of resources spent on general palliative care during the last decade. The reported clinical practice for screening for life-threatening diseases and assessment of the need for palliative care was associated with the type of specialty and the attitude of the department and in the multivariable analyses, not the department size, which otherwise might impact leadership complexity and priorities. The regions had no impact on the clinical practice either, which is surprising since the regional management determines the political priorities of hospitals and departments and since the regions have varying population characteristics and rural/urban composition.

### What this study adds

To our knowledge, this nationwide mapping of general palliative hospital care is the first of its kind. A nationwide study is made possible by the public healthcare system, national recommendations and one single official language – conditions that could make nationwide surveys difficult if not met [15]. This survey described that physical symptoms are relieved more often than spiritual problems and that palliative measures in all dimensions are carried out to a slightly greater extent than documented. A recent scoping review [16] examined how rehabilitation and palliative care approaches were combined in interventions for patients with life-threatening illnesses and the findings were that social and spiritual dimensions were rarely evaluated, which is in line with results from the survey in this study. This suggests that fostering a culture that values addressing all aspects of palliative care is crucial for holistic palliative care. Furthermore, palliative measures are delivered more often than documented, which may impact continuity of care and appropriate remedy.

The results from this study show that the most common method for assessing palliative care needs is a general impression of the patient’s condition followed by open-ended questions. While these can be helpful in starting conversations, they might underestimate symptoms. The median number of symptoms which can be found using systematic assessment can be tenfold higher than open-ended questioning [17].

This study demonstrated an association between a department’s attitude toward using screening tools and conducting needs assessments and their reported practices of planning end-of-life care with patients. This result reinforces the importance of leadership buy-in for patient-centered approaches. A significant barrier to wider utilization of palliative care has been shown to lie in stereotypes about palliative care held by some healthcare providers [18]. Understanding these prevailing attitudes is crucial for the successful design and implementation of a systematic palliative care programs as suggested by the theoretical domains framework [19].

The results highlight a potential link between a department chief’s attitude towards palliative care and the department’s clinical practices. The fact that factors like region, department size (inpatient admissions), and outpatient volume had little impact on the results suggests that the leadership’s perspective can significantly influence department practices in general palliative care. Thus, training and education for the department chiefs may improve departmental practices and ensure patients receive appropriate care throughout their illness.

One-quarter of the responding departments reported always or often screening for life-threatening diseases. In some departments, screening may be irrelevant if the patient’s disease status is obvious. Still, the result may indicate missed opportunities for early intervention and timely initiation of palliative care, supported by the median survival time in specialized palliative care, which is 19 days across countries [20].

The results from this study also indicate a lack of clear ownership for initiating end-of-life care planning. High percentages of responsibility across multiple groups indicates no single party in the health care system is perceived as responsible for bringing up end-of-life planning. Diffusion of responsibility is known to impede action where multiple actors share accountability [21, 22]. The phenomenon thrives in fragmented healthcare systems and may result in no-one initiating end-of-life care planning. Furthermore, the results show that 57% view the patient as responsible for initiating these discussions, highlighting the importance of empowering patients to participate in end-of-life care planning. The respondents identified general practitioners as the second most responsible party for initiating end-of-life care planning. General practitioners may enable palliative care by taking a holistic, patient-centered approach and providing continuous care [23]. Future developments could include task division between primary, secondary and tertiary care to avoid diffusion of responsibility.

### Strengths and limitations

The study was a national survey with an excellent response rate, and the results may be generalizable to other countries with similar healthcare systems. The profile of non-responders may differ from responders, which could potentially affect the results, although we are not aware of any empirical studies to support speculation in how non-responders would have responded. The questionnaire had not undergone a full psychometric evaluation, but the development process included cognitive interviewing supporting face validity. Responders dropped out of questionnaire completion at different items, indicating that that dropout was not promoted by particular items. The size of departments varied greatly, and some department heads may lack insight into the daily clinical practice described in some items. Potentially, first-line managers or clinical employees might have responded differently to the survey with closer insight into day-to-day clinical care but without the overview of the entire department and without insight into resources used. However, the maximum proportion of ‘unsure’-responses was 19%, which indicates a high level of perceived knowledge about clinical practice. Respondents were instructed to gather information from employees if needed to answer the survey.

The number of completed questionnaires set the limits for details in the logistic regression analyses. Outcomes and explanatory variables were dichotomized, and the types of specialties had an ‘other and mixed’ group of departments with mixed surgical and medical specialties together with emergency, psychiatry, and others. Nuances and details were invariably lost in the process.

The questionnaire was intended to assess the viewpoints of the department heads as representatives of the whole department, and the results are, therefore, limited to a conclusion on self-reported practice and not the quality of patient care. A self-report assessment of content indicating quality of care could potentially be overestimated.

The 10-year increase could potentially be ascribed to an increased focus on palliative care since the healthcare system generally has fewer resources in that time period. A systematic review was unable to identify any models for inpatient general palliative care and seven ambulatory clinic interventions with mixed populations and results [24]. With limited evidence to recommend specific models of general palliative care, it is unknown whether the 10-year increase in prioritizing resources translates to improved clinical effect.

This study explored the association between attitudes towards palliative care tasks and clinical practice. Other factors that might affect general palliative care could be staff skill mix and vacancies which were not available in the data set.

### Perspectives

The study results could serve as benchmarks for the development of quality indicators for general palliative care and further policy development. The rare use of well-defined methods for target group identification and needs assessment calls for the development of clinical guidelines as well as organizationally sustainable care pathways to ensure healthcare equity. Furthermore, the significance of attitudes calls for training directed at department heads as well as clinical staff.

## Supplementary Information

Supplementary Material 1.

## Data Availability

The datasets generated and analyzed during the current study are not publicly available due to participant confidentiality but are available from the corresponding author on reasonable request after anonymization.
