# Supplementary material for: General palliative hospital care – a Danish nationwide survey of organization and clinical practice
Source: BMC Palliat Care. 2026 Jan 28;25:49. doi: 10.1186/s12904-026-02005-3 (PMC12924260; doi:10.1186/s12904-026-02005-3)
Supplement: Supplementary file 1 — Supplementary Material 1. [file 12904_2026_2005_MOESM1_ESM.docx]

# Supplementary

**Figure S1. Flowchart**


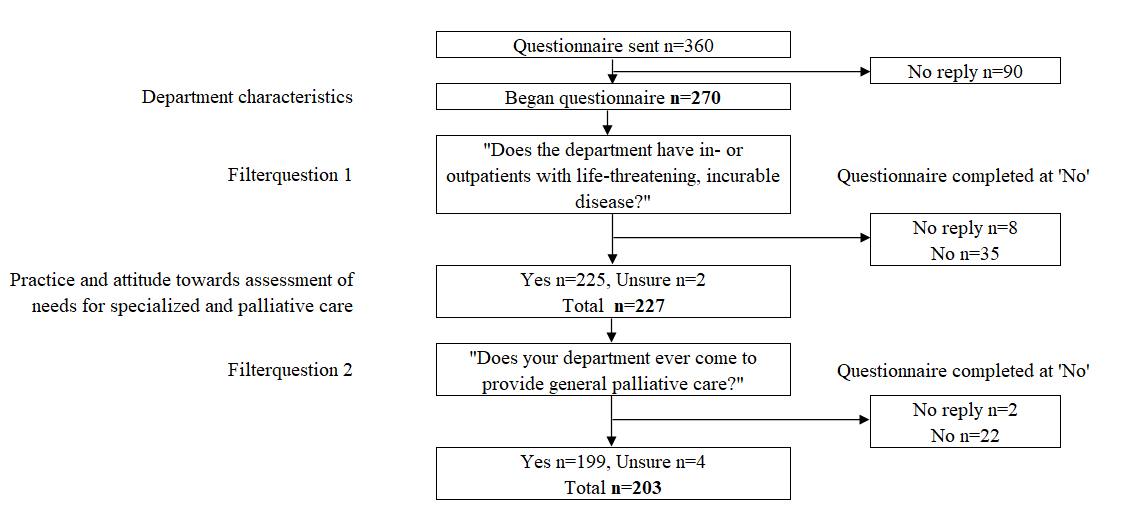


**Table S1. Characteristics of participating departments**

| Professional background of responder (n=270) | n |
| --- | --- |
| Physician | 123 |
| Nurse | 140 |
| Other | 7 |
| Specialties (n=264) * |  |
| Anaestesiologi and intensive care | 36 |
| Cardiology | 26 |
| Endocrinology | 23 |
| Gastroenterology and hepatology | 22 |
| Geriatrics | 22 |
| Surgery | 22 |
| Pulmonary medicine | 22 |
| Orthopedic surgery | 22 |
| Pediatrics | 22 |
| Emergency medicine | 21 |
| Gynaecology and obstetrics | 21 |
| Psychiatry | 21 |
| Neurology | 18 |
| Rheumatology | 16 |
| Nephrology | 15 |
| Infection medicine | 14 |
| Oncologi | 14 |
| Urology | 13 |
| Haematology | 11 |
| Specialized palliative care** | 10 |
| Other (< 10) | 36 |
| Hospital admissions per year (n=262) |  |
| Less than 2000 | 122 |
| 2000+ | 103 |
| Unknown | 37 |
| Outpatient contacts per year (n=262) |  |
| Less than 10.000 | 100 |
| 10.000+ | 135 |
| Unknown | 27 |
| *Some departments have more than one specialty, ** Respondents were instructed to exclude information from specialized palliative care and focus on general palliative care |  |

**Table S2. Association between type of specialty and palliative care practices**

| **Outcome** | **Explanatory variable** | **n** | **Crude** | | **Adjusted*** | |
| --- | --- | --- | --- | --- | --- | --- |
|  |  |  | **OR** | **95% CI** | **OR** | **95% CI** |
| Use of screening tools | Medical | 73 | Ref | | Ref | |
|  | Surgical | 32 | 0.265 | (0.097 - 0.719) | 0.253 | (0.088 - 0.730) |
|  | Other and mixed | 90 | 0.574 | (0.304 - 1.083) | 0.524 | (0.267 - 1.031) |
| Assessment of needs for general palliative care | Medical | 77 | Ref | | Ref | |
|  | Surgical | 33 | 0.425 | (0.185 - 0.976) | 0.413 | (0.171 - 0.999) |
|  | Other and mixed | 103 | 0.909 | (0.489 - 1.691) | 0.984 | (0.514 - 1.883) |
| Assessment of needs for specialized palliative care | Medical | 76 | Ref | | Ref | |
|  | Surgical | 33 | 0.467 | (0.190 - 1.146) | 0.410 | (0.152 - 1.105) |
|  | Other and mixed | 103 | 0.476 | (0.240 - 0.942) | 0.448 | (0.215 - 0.933) |
| Planning end-of-life care together with the patient | Medical | 72 | Ref | | Ref | |
|  | Surgical | 31 | 0.934 | (0.397 - 2.195) | 0.861 | (0.348 - 2.130) |
|  | Other and mixed | 87 | 0.911 | (0.483 - 1.719) | 0.957 | (0.492 - 1.862) |
| *Adjusted for region, yearly inpatient admissions, and yearly outpatient contacts | | | | | | |

Table S2 demonstrates the association between type of specialty and the reported use of tools for target group assessment, palliative care needs assessment, and planning the end of life together with the patient.

**Figure S2. The relief and documentation of the four dimensions of palliative care**

*More than one response was allowed, and the total is more than 100%. There were 5 missing responses.
